# Intercropping of wheat and walnut reduce populations of *Chromaphis juglandicola*

**DOI:** 10.1093/jee/toaf110

**Published:** 2025-07-30

**Authors:** Shuangshuang Jia, Yuyang Shen, Feifei Deng, Jin Li, Guangkuo Li, Haifeng Gao, Yongqiang Liu

**Affiliations:** State Key Laboratory for Biology of Plant Diseases and Insect Pests, Institute of Plant Protection, Chinese Academy of Agricultural Sciences, Beijing, China; Institute of Plant Protection, Xinjiang Academy of Agricultural Sciences, Key Laboratory of Integrated Pest Management on Crop in Northwestern Oasis, Ministry of Agriculture and Rural Affairs, Urumqi, China; Institute of Plant Protection, Xinjiang Academy of Agricultural Sciences, Key Laboratory of Integrated Pest Management on Crop in Northwestern Oasis, Ministry of Agriculture and Rural Affairs, Urumqi, China; Institute of Plant Protection, Xinjiang Academy of Agricultural Sciences, Key Laboratory of Integrated Pest Management on Crop in Northwestern Oasis, Ministry of Agriculture and Rural Affairs, Urumqi, China; Institute of Plant Protection, Xinjiang Academy of Agricultural Sciences, Key Laboratory of Integrated Pest Management on Crop in Northwestern Oasis, Ministry of Agriculture and Rural Affairs, Urumqi, China; Institute of Plant Protection, Xinjiang Academy of Agricultural Sciences, Key Laboratory of Integrated Pest Management on Crop in Northwestern Oasis, Ministry of Agriculture and Rural Affairs, Urumqi, China; State Key Laboratory for Biology of Plant Diseases and Insect Pests, Institute of Plant Protection, Chinese Academy of Agricultural Sciences, Beijing, China

**Keywords:** agricultural intensification, intercropping, pest biological control, predators

## Abstract

Intensive agriculture is associated with a great decline in biodiversity and biocontrol of pests. Intercropping potentially promotes natural enemy’s abundance and species biodiversity and enhance pest biocontrol. We monitored the population dynamic of *Chromaphis juglandicola* (Kaltenbach) (Hemiptera: Aphididae) and tested the effect of intercropping conventionally managed winter wheat with walnut on the abundance of *C. juglandicola* and its predators (including ladybeetles, lacewings, hoverflies, and spiders), and the diversity, evenness, and dominant concentration of predators on walnut. The density of *C. juglandicola* in walnut trees was peaked in mid-July. It, but not its predators, was significantly reduced in walnut–wheat plots, but the species diversity and evenness of predators declined, and the dominant concentration increased. Intercropping benefited *C. juglandicola* control by reducing their density in walnut. Overall, the increasing of plant diversity in intercropping system of wheat with walnut enhance the aphid *C. juglandicola* biocontrol, which can reduce the reliance on pesticide in walnut and result in a walnut producing agroecosystem that is more nature-inclusive and healthy for human and the ecosystem.

## Graphical Abstract

**Graphical Abstract:**
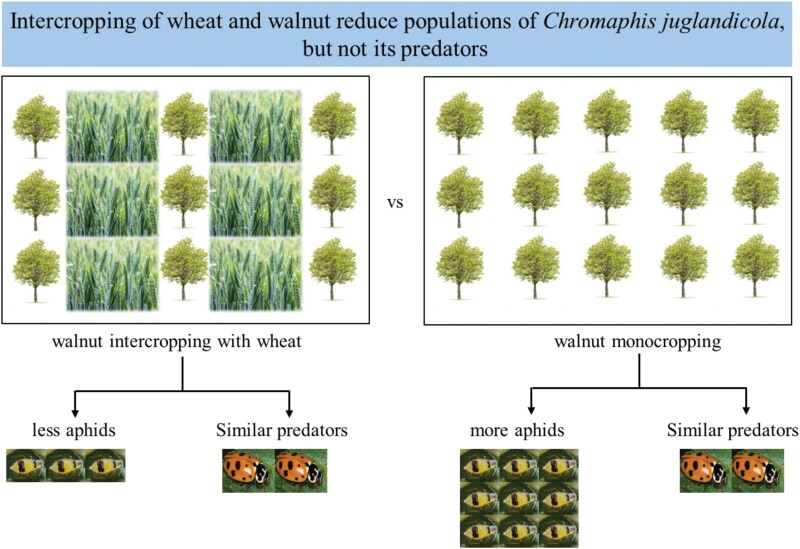

## Introduction

Agricultural intensification, characterized by intensive field management and large areas of single crops, is widely adopted throughout the world (Rosenheim et al. 2023) to meet the demands and desires of the growing world population (Kessler and Baldwin 2001). However, such intensification is accompanied by dramatic declines in biodiversity and impaired ecosystem services, such as natural biocontrol of insect pests (Lichtenberg et al. 2017, Zou et al. 2019, Bakker et al. 2021). As a result, more insecticides are applied, increasing the risks to the environment, ecosystem service, and human health (Paredes et al. 2021). Thus, more sustainable production systems that support abundance and species diversity of natural insect enemies, improve ecosystem services, and increase field productivity are urgently needed.

Biological control of agricultural pests is environmentally sound and effective (Pei et al. 2018, Dainese et al. 2019) and promoted by diversified agroecosystems that help maintain a biodiverse insect population (Iverson et al. 2014, Wan et al. 2020). Manipulative experiments broadly suggest that increasing natural enemy species diversity and evenness generally strengthens pest biological control when enemy species complement one another by occupying different feeding niches. Intercropping, that is, growing 2 or more crops at the same time, has been widely adopted to promote diversification for sustainable agriculture throughout the world, especially in developing countries (Vandermeer 1992, Brooker et al. 2015, Arsyad et al. 2020).

Intercropping contributes to pest biocontrol through various mechanisms, including “bottom-up” and “top-down” effects (Peter 1993, Ju et al. 2019, Huss et al. 2022). For a “bottom-up” effect, planting a diversity of crops may hamper host foraging by acting as a physical barrier that impedes visual searching and hinders olfactory orientation by the pests (Kessler and Baldwin 2001, Mansion‐Vaquié et al. 2019, Tooker et al. 2020). In a “top-down” effect, intercropping can provide alternative prey or plant-based food (eg pollen and nectar) or shelter to enhance the abundance and diversity of natural enemies (Wäckers et al. 2005, Gurr et al. 2017, Gontijo 2019). However, increased biodiversity of crops does not always benefit pest biocontrol; some intercropping systems have no effect on the biocontrol of pests or even promote pests (Li et al. 2020, 2021, Flausino et al. 2022).

In the southern Xinjiang Uygur Autonomous Region (Xinjiang) in China, intercropping has been implemented for many years (Peichl et al. 2006, Feike et al. 2010, Hong et al. 2020). Although this area has low rainfall, the light and thermal regime support the rapid growth of fruit and nut trees, which produce good-quality harvests. To maximize the use of resources and increase yields and economic benefits, farmers have developed unique production systems using fruit or nut trees intercropped with crops (Wang et al. 2010, Li et al. 2018). One of the most important of these systems in southern Xinjiang is walnut intercropped with wheat.

In walnut, the aphid *Chromaphis juglandicola* (Kaltenbach) (Hemiptera: Aphididae) is the most harmful pest. Its control depends mainly on chemical pesticides, which may result in pesticide treadmill, the vicious cycle that farmers increase pesticide use to control pests due to resistance and the lethal or sublethal effect of pesticide to natural enemy (Cowan and Gunby 1996, Bakker et al. 2020). A detailed understanding of the occurrence and dynamics of this pest is fundamental to its control period. Wheat sown in October and harvested in July of the following year. Wheat supports aphid populations and receives only few insecticide applications and can therefore support natural enemies and may influence pest suppression in other surrounding crops when the natural enemies move out from wheat to other fields (Ouyang et al. 2012, 2020, Zhou et al. 2014). However, no studies have elucidated the population dynamics of *C. juglandicola* or predator in wheat–walnut intercropping and walnut monoculture system, and how wheat–walnut intercropping influence the species diversity, evenness, and dominant concentration of predators. In the present study, we collected data on the abundance of *C. juglandicola* and its predators to describe the population dynamics of the insects on walnut in wheat–walnut intercropping and walnut monoculture systems in southern Xinjiang, China. The goal of our study was to (i) identify the effects of the intercropping on the species diversity, evenness, and dominant concentration of natural predators, (ii) elucidate the population dynamics of *C. juglandicola* and its predators, and (iii) investigate the effects of wheat–walnut intercropping on the density of *C. juglandicola* and its predators in walnut. This information will provide insight regarding the influence of wheat–walnut intercropping on pest biocontrol, which may lead to an eco-friendly management strategy.

## Materials and Methods

### Study Area

Experiments were carried out in 2018 in Zepu County (37°57′N~38°19′N, 76°52′E~77°29′E), Kashgar, Xinjiang Uygur Autonomous Region, China. In the Köppen climate classification, this region has a cold desert climate (BWk) and cold steppe climate (BSk) with a median yearly annual precipitation of 240 mm (Kottek et al. 2006). Walnut, wheat, jujube, apple, cotton, and maize are the main crops, and fruit–grain intercropping is common. The walnut trees were 8 to 10 yrs old. The conventional management of wheat and walnut trees in the region includes one insecticide spray at the end of April, regular drip irrigation from April to August, and manual weeding at the end of May. Wheat is sown in October the year before and harvested in July the following year. The growing season of wheat and walnut overlap from April to July.

### Experimental Design

**Fig. 1.**
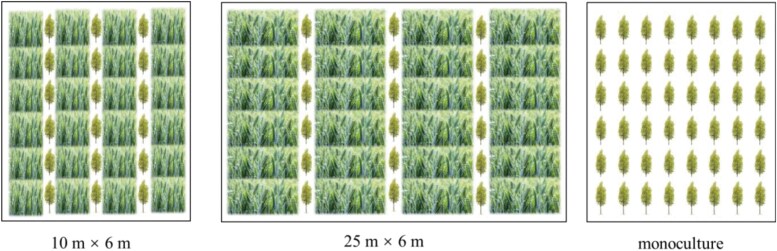
Diagram of width of rows in wheat–walnut intercropping systems and walnut monoculture: 10 m wheat intercropped with 6 m walnut trees (10 × 6 m; left), 25 m wheat intercropped with 6 m walnut trees (25 × 6 m; middle), and walnut monoculture (monoculture; right).

Wheat was grown between the rows of walnut trees. The intercropping strategy for our study consisted of 3 levels: a 10-m-wide row of wheat intercropped with a 6-m-wide row of walnut trees (10 × 6 m), 25-m-wide row of wheat intercropped with 6-m-wide row of walnut trees (25 × 6 m), and a monoculture of walnut (Fig. 1). We chose the wheat–walnut intercropping patterns of 10 × 6 m and 25 × 6 m pattern because they were widely adopted by local farmers. Four villages were selected as 4 replicates with a distance of at least 2 km between them. Three plots, at least 1 ha each, were set up in each of the 4 replicates for a total of 12 plots (3 plots × 4 replicates). The area of each plot was at least 100 × 100 m. The variety of wheat and walnut were Xindong 20 and Wen 185, respectively, which are common in this area. The space between 2 adjacent wheat rows was 15 cm and between 2 adjacent walnut trees in a walnut row was 6 m. According to conventional management by local farmers, wheat in this study was sown in October the year before and harvested in July the following year.

### *C. juglandicola* and Predator Survey

For 6 walnut trees in the center of each field, we examined a 30-cm-length at the end of 2 (upper and lower layers) branches in each direction (east, west, north, and south) and recorded the number of *C. juglandicola* and its predators. Insects were identified on the branches without removing them. *C. juglandicola* and predators were classified by morphological characteristics. When recording observational data, we classified predators by family. We surveyed the trees once a week from 8 April to 26 August, which spans the entire growth period of walnut trees in a season.

### Statistical Analyses

The Shannon–Wiener diversity index (*H*) was calculated as $H=-\overset{s}{\underset{i=1}{\sum }}P_{i}ln\;P_{i}$. The evenness index (*J*) was calculated as J=(H/ln⁡ S) (Wu et al. 2017). Dominant concentration index (*C*) was calculated as $C=\sum_{i=1}^{S}{\left(\frac{N_{i}}{N}\right)}^{2}=\sum_{i=1}^{S}{\left(P_{i}\right)}^{2}$ (Wu et al. 2017), where *S* is the number of predator families, *P*_*i*_ is the proportion of family *i* in the total number of all predators in the population. *N* is the total number of individuals of predators, and *N*_*i*_ is the number of individuals of predator family *i*. The 3 index were calculated using the pooled number of each predator family sampled in each replicate over the whole season.

Kruskal–Wallis test was used to analyze the Shannon–Wiener diversity index, species evenness index, and dominant concentration for differences among the 3 cropping treatments. There were 4 replicates (4 plots) for each treatment. The Shannon–Wiener diversity index, species evenness index, and dominant concentration were treated as response variables, and treatment (10 × 6 m, 25 × 6 m, and monoculture) was treated as the explanatory variable. Dunn’s multiple comparison test was used to detect differences between any 2 pairs of the 3 treatments.

We used a generalized linear mixed models (GLMM) with negative binomial error distribution to analyze the data of *C. juglandicola* and its predators. The response variables were population density of *C. juglandicola* or predators per replicate. We used treatment (10 × 6 m, 25 × 6 m and monoculture) as fixed effect. Replicate (4 replicates) and sampling date were included as crossed random effects (*C. juglandicola* or predators ~ Treatment + (1|replicate) + (1|sampling date)).

R version 4.2.1 (R Core Team 2020) was used for all analyses. Graphs were made in GraphPad Prism (Motulsky 1989).

## Results

### Shannon–Wiener and Evenness Indices and Dominant Concentration of Predators

**Fig. 2.**
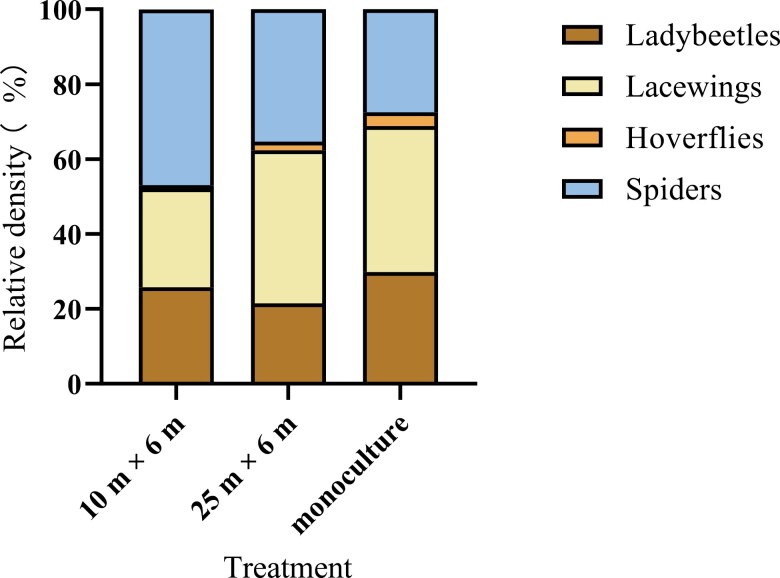
The percentage of different predators in the 3 field treatments (wheat–walnut intercropping 10 × 6 m, wheat–walnut intercropping 25 × 6 m, and walnut monoculture).

In total, we sampled 6,212 predators in walnut trees in the 10 × 6 m treatment, 5,077 predators in walnut trees in the 25 × 6 m treatment, and 7,834 predators in walnut trees in walnut monoculture treatment. Predators included ladybeetles (Coleoptera: Coccinellidae), *Adonia variegate* (Goeze), *Propylaea japonica* (Thunberg), *Propylaea quatuordecimpunctata* (Linnaeus), *Oenopia conglobate* (Linnaeus), and *Coccinella undecimpunctata* (Linnaeus), lacewings (Neuroptera: Chrysopdidae): *Chrysopa carnea* (Stephens); hoverflies (Diptera: Syrphidae): *Eupeodes corollae* (Fabricius) and *Sphaerophoria scripta* (Linnaeus), and spiders. In the 10 × 6 m treatment, 1,602 ladybeetles, 1,639 lacewings, 53 hoverflies, and 2,918 spiders were sampled (25.79%, 26.38%, 0.85%, and 46.97% of the total, respectively). In the 25 × 6 m treatment, we found 1,091, 2,076, 117, and 1,793 individuals in these respective families, respectively (21.49%, 40.89%, 2.30%, and 35.32% of the total, respectively). In the walnut monoculture, their abundance was 2,341, 3,046, 296, and 2,151, respectively (29.88%, 38.88%, 3.78%, and 27.46% of the total, respectively) (Fig. 2).

**Fig. 3.**
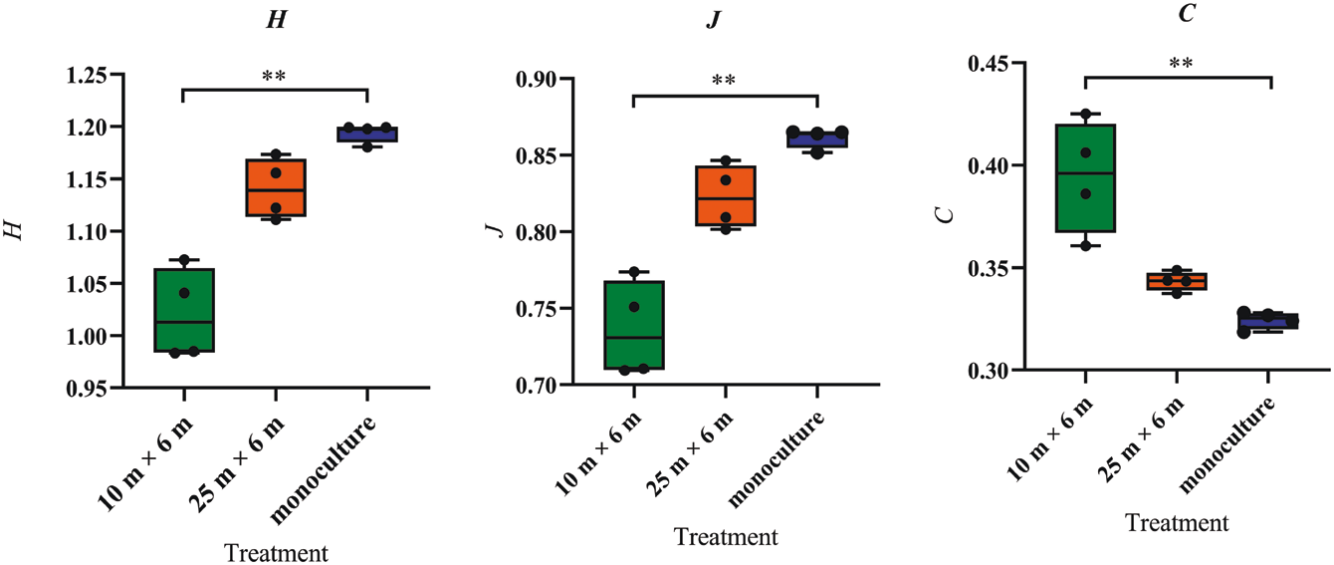
Box plots and result of Dunn’s multiple comparison test of Shannon–Wiener index (*H*), evenness index (*J*), and Dominant concentration (*C*) of predators in the 3 field treatments (wheat–walnut intercropping 10 × 6 m, wheat–walnut intercropping 25 × 6 m, and walnut monoculture). Significance levels: *P < 0.05; **P < 0.01; ***P < 0.001.

The Shannon–Wiener (*H*) index (*χ*^2^ = 9.85, df = 2, *P* < 0.01), the evenness (*J*) index (*χ*^2^ = 9.85, df = 2, *P* < 0.01), and dominant concentration (*C*) (*χ*^2^ = 9.85, df = 2, *P* < 0.01) were significantly influenced by treatments. Both the Shannon–Wiener (*H*) index and the evenness (*J*) index for the 10 × 6 m intercropped plot was lower than for the walnut monoculture (*P* < 0.01, *P* < 0.01) and no significant difference of *H* or *J* was found between the 10 × 6 m and 25 × 6 m intercropped plot or between the 25 × 6 m intercropped plot and monoculture plot (Fig. 3A and B). On the contrary, dominant concentration (*C*) for the 10 × 6 m intercropped plot was higher than for the walnut monoculture (*P* < 0.01) and no significant difference of *C* was found between any other 2 treatments (Fig. 3c).

### Populations Dynamics: C. juglandicola and Predators

**Fig. 4.**
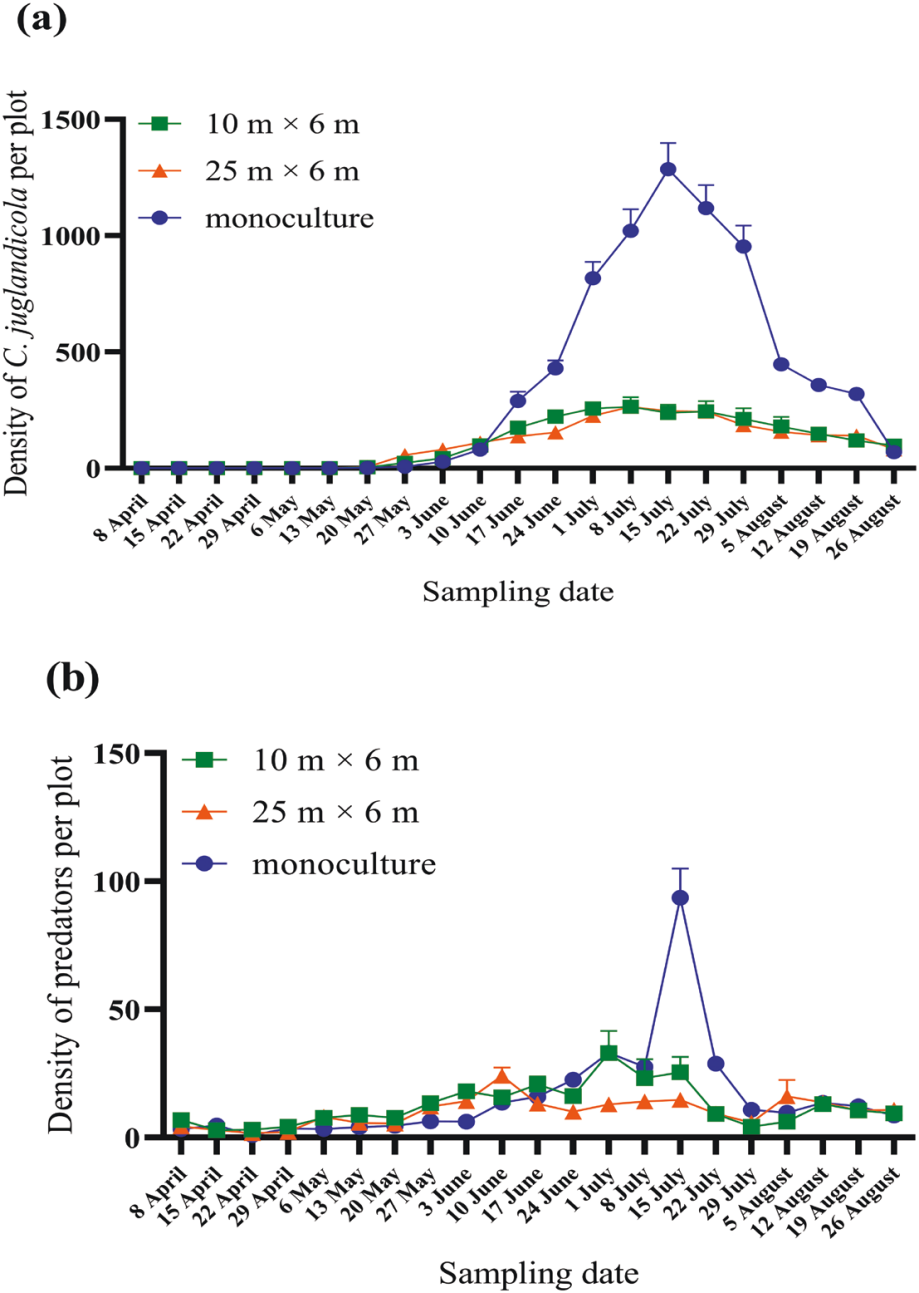
Population dynamics (density per plot) of *C. juglandicola* (A) and its predators (B) from early April to the end of August in the 3 treatments (wheat–walnut intercropping 10 × 6 m, wheat–walnut intercropping 25 × 6 m, and walnut monoculture).

The density of *C. juglandicola* in walnut trees remained low (less than 100 per plot) before June in all 3 treatments. From early June, its population in the monoculture grew rapidly and peaked (around 1,200 per plot) in mid-July. Then its population declined rapidly to less than 100 per plot by the end of August. The overall population dynamics of *C. juglandicola* in walnut trees in the 2 intercropped plots and the monoculture were similar: increasing from late May until peaking in July, then decreasing. However, the peak density of *C. juglandicola* in walnut trees in the 10 × 6 m and in the 25 × 6 m intercropped plot was more than 5 times lower than in the monoculture (Fig. 4A).

The predator population dynamics in walnut was similar to that of *C. juglandicola*. Before June, the abundance of predators in walnut in the 3 treatments was less than 10 per plot. From early June, in the monoculture, the predator density peaked (nearly 100 per plot) in mid-July, then declined. Predator abundance in the intercropped walnut began to increase in late May then fluctuated somewhat through the rest of the season without having an obvious peak. The population was highest (approximately 15 to 30 per plot) in June and July but only slightly (Fig. 4B).

### Population Density Comparison: C. juglandicola and Predators

The results of GLMM analysis indicated that the total density of *C. juglandicola* per plot from early April to the end of August in the walnut monoculture was significantly higher than in the 10 × 6 m (*P* < 0.001) and the 25 × 6 m (*P* < 0.001) intercropped plots (Table 1). The total density of predators per plot from early April to the end of August in the walnut monoculture was significantly higher than in the 25 × 6 m intercropped plot (*P* < 0.05) but not in the 10 × 6 m plot(*P* > 0.05; Table 1).

**Table 1.** Results of a GLMM with negative binomial error distribution to compare the density of *C. juglandicola* and predators observed on walnut trees in 3 treatments over 4 replicates and all sampling dates

Population	Effects	Estimate	Std. Error	*z* value	Pr (>|*z*|)
*C. juglandicola*	Intercept	5.022	0.803	6.251	<0.001***
	Treatment 10 m:6 m	−0.586	0.116	−5.058	<0.001***
	Treatment 25 m:6 m	−0.546	0.118	−4.652	<0.001***
Predators	Intercept	4.130	0.178	23.173	<0.001***
	Treatment 10 m:6 m	−0.009	0.092	−0.096	0.924
	Treatment 25 m:6 m	−0.185	0.093	−1.981	0.048*

*C. juglandicola* and predators in the monoculture treatment was the reference. Significance levels: * *P* < 0.05; ** *P* < 0.01; *** *P* < 0.001.

## Discussion

We monitored the population dynamics of *C. juglandicola* and its predators in walnut in 2 wheat–walnut intercropping systems and the walnut monoculture and analyzed the influence of wheat–walnut intercropping on the abundance of *C. juglandicola* and abundance and diversity of the predators. We found that wheat–walnut intercropping reduced aphid pressure compared with the monoculture. *C. juglandicola* density decreased by about 80% in walnut trees in the intercropped plots in the whole season. Predator abundance, however, was not higher in the intercropped plots than in the monocultures, and the species diversity and evenness decreased, but the dominant concentration increased.

Understanding the population dynamics of *C. juglandicola* and its predators provides guidance for developing strategies to control *C. juglandicola*. Because populations of *C. juglandicola* in monoculture walnut increased rapidly from early June, peaked in mid-July, and then decreased, measures should be taken before June to keep the population density low and limit damage during the peak outbreak. However, the predator population in monoculture walnut did not increase greatly with the outbreak of *C. juglandicola*; thus, growers cannot rely only on natural enemies to control the aphids. Other strategies such as growing aphid-resistant varieties, applying appropriate pesticides at a suitable dose time also need to be included to control aphids.

The benefits of intercropping on pest biocontrol and biodiversity have been extensively documented (Hatt et al. 2017, Ribeiro and Gontijo 2017, Zheng et al. 2020, Hunt et al. 2021), while neutral or even negative impacts of intercropping have also been reported (Kruidhof et al. 2015, Flausino et al. 2022). As we had hoped, aphid density in the intercropped plots was lower than in the walnut monoculture, but predator density and the species diversity of the predators was not higher. However, the reason for this finding is not clear. We hypothesized that the fewer aphids on intercropped walnut trees could not attract a wider variety of predators. For example. Coccinellids prefer to lay eggs on infected plants with large number of aphids (Banks 1956). But this hypothesis cannot be tested in our study. The importance of natural enemies for pest control by a “top-down” process has been verified by numerous studies (Mills 2001, Haan et al. 2020, Almdal and Costamagna 2023). In another study focusing on aphid and predator abundance on wheat, we reported larger aphid and predator populations on wheat in wheat–walnut intercropping system. The more predators supported by wheat in this intercropping system may have suppressed aphid on walnut (Gao et al. 2024). However, a decrease in the pest population is not always correlated with an increase in their predators. Almdal et al. (2023) also found that planting diverse crops help to suppress aphid population density during outbreaks but does not impact the predator population. The reduced pest density may be due mainly to a “bottom-up” process (Almdal and Costamagna 2023). In an intercropping system, the presence of a nonhost crop may impede the ability of pests to locate a host plant (Mansion‐Vaquié et al. 2019). Because *C. juglandicola* is monophagous (Bozenna and Katarzyna 2007), interplanting wheat between 3 rows of walnut trees could make finding the host more difficult, resulting in fewer aphids in the trees.

The abundance of aphids is also closely related to the microclimate. High temperature, low humidity, and high wind speed, as found in our study area in spring and summer, lower aphid incidence (Wang et al. 2016). However, the shade provided by walnut trees may also provide a suitable microclimate for the aphids with lower temperature, higher humidity, and lower wind speed. Compared to walnut monoculture, rows of wheat between walnut trees in wheat–walnut intercropping fields may have their own microclimate, with higher temperatures, lower humidity, and faster winds that are less conducive to aphid survival and reproduction than walnut monocultures. At the same time, this adverse microclimate in wheat strips may also have a neutralizing effect on the relative favorable environment of walnut trees, leading to lower aphid density in walnut in wheat-walnut intercropping fields.

As mentioned earlier, the lower density of aphids in the intercropped treatments compared with the monocultures was not due to an increase in predators; predator density in the intercropped plots was not lower than the monoculture. On the contrary, the total ladybeetle density in walnut in the 25 × 6 m intercropped plot was lower than in the walnut monoculture. Because prey density is the main factor that determines the population size of predators, predator abundance is not likely to be higher when wheat is present between walnut rows because the prey abundance is lower and could explain why ladybeetle density was not higher in the intercropped plot than in the monoculture. In our earlier study, however, we also found more aphid individuals [*Sitobion avenae* (Fabricius), Hemiptera: Aphididae] on wheat plants in the intercropped plots that could provide prey for predators and might attract predators from the walnut trees (Gao et al. 2024). At the same time, aphid on wheat plants also nourished predators and enhanced predator abundance in the intercropping systems. This in turn helps aphid control in walnut trees.

By elucidating the population dynamics of *C. juglandicola* and its predator in walnut from April through August, we now have a guide for aphid control in walnut using intercropping with wheat. We also provided evidence that intercropping reduced the pest population below that in the monoculture, highlighting the benefits of this diversified agroecosystem. We did not find evidence that intercropping improved the density or species diversity of the predators. However, we still need to study the influence of the wheat–walnut intercropping system on other pests, natural enemies, biodiversity, and yield before the system can be adopted for practical use.
